# Altered Modular Organization of Functional Connectivity Networks in Cirrhotic Patients without Overt Hepatic Encephalopathy

**DOI:** 10.1155/2014/727452

**Published:** 2014-06-05

**Authors:** Gang Zheng, Liping Zhang, Long Jiang Zhang, Qiang Li, Zhiying Pan, Xue Liang, Donghong Shi, Guang Ming Lu

**Affiliations:** ^1^Department of Medical Imaging, Jinling Hospital, Clinical School of Medical College, Nanjing University, Nanjing, Jiangsu 210002, China; ^2^College of Civil Aviation, Nanjing University of Aeronautics and Astronautics, Nanjing, Jiangsu 210016, China; ^3^College of Natural Science, Nanjing University of Aeronautics and Astronautics, Nanjing, Jiangsu 210016, China

## Abstract

Minimal hepatic encephalopathy (MHE) is associated with changes in functional connectivity. To investigate the patterns of modular changes of the functional connectivity in the progression of MHE, resting-state functional magnetic resonance imaging was acquired in 24 MHE patients, 31 cirrhotic patients without minimal hepatic encephalopathy (non-HE), and 38 healthy controls. Newman's metric, the modularity *Q* value, was maximized and compared in three groups. Topological roles with the progression of MHE were illustrated by intra- and intermodular connectivity changes. Results showed that the *Q* value of MHE patients was significantly lower than that of controls (*P* < 0.01) rather than that of non-HE patients (*P* > 0.05), which was correlated with neuropsychological test scores rather than the ammonia level and Child-Pugh score. Less intrasubcortical connections and more isolated subcortical modules were found with the progression of MHE. The non-HE patients had the same numbers of connect nodes as controls and had more hubs compared with MHE patients and healthy controls. Our findings supported that both intra- and intermodular connectivity, especially those related to subcortical regions, were continuously impaired in cirrhotic patients. The adjustments of hubs and connector nodes in non-HE patients could be a compensation for the decreased modularity in their functional connectivity networks.

## 1. Introduction

The human brain can be regarded as a complex network, which is organized intrinsically as highly modular architectures with inter- and intramodular links between brain regions [1–5]. The modules or communities of a complex network are subsets of nodes [4, 6–8]. Modularity in the human brain has been identified by both structural and functional MRI studies [1, 2]. Detection and characterization of modular structure in the brain system can help identify groups of anatomically and/or functionally associated components performing specific biological functions [3]. Modular structure is crucial for the robustness of network stability and optimal network functions [9], and modular structure is related to the balance of functional segregation and integration and high resilience to network node or edge damage. It has been shown that modularity of brain networks may play a critical role in the evolution and neurodevelopment [2]. Some studies have shown the disruptions of functional brain network modularity in patients with childhood-onset schizophrenia [10], schizophrenia [11, 12], epilepsy [13, 14], and chronic back pain [15].

Hepatic encephalopathy (HE) is a serious neuropsychiatric complication of both acute and chronic hepatic dysfunctions [16], which is characterized by a wide clinical spectrum, ranging from mild cognitive impairment to coma and death. Minimal hepatic encephalopathy (MHE), the mildest form of the spectrum of HE, usually has no recognizable clinical symptoms of HE but has mild cognitive, motor control, and concentration attention deficits [17, 18]. In recent years, the diagnosis, pathophysiological mechanisms, and treatment of MHE have drawn wide attention. Many functional MRI (fMRI) studies have been performed to clarify the pathophysiological mechanisms of MHE. Some fMRI studies focus on the analysis of regional brain networks, supporting that the regional functional connectivity modules (e.g., cognitive, motor control, and concentration attention) of cirrhotic patients were impaired [19–21]. Different from those studies based on regional brain networks, Zhang et al. investigated patterns of whole-brain functional connectivity in cirrhotic patients with MHE and found widespread cortical and subcortical network connectivity changes, suggesting that not only functional connectivities within regions but also those between functional modules were impaired in MHE patients [22]. In particular, the impairment in the basal ganglia-thalamocortical circuit was found which could play an important role in mediating neurocognitive dysfunctions, especially for psychomotor speed and attention deficits in patients with MHE [22, 23, 37]. However, it is still unclear how functional connectivity within and between modules changes during the progression of MHE.

Based on the previous findings of widespread decreased cortical and subcortical network connectivity in MHE patients, we hypothesize that the community structure itself, including inter- and intramodular links between brain regions, is impaired in MHE patients, and functional connectivity change in the modular organized brain is associated with the development of MHE. The aim of this study was to quantitatively evaluate the modularity changes of functional connectivity network in healthy subjects, cirrhotic patients without minimal HE (non-HE), and MHE patients and to illustrate the patterns of spatial change of modular community structure in the development of MHE. Additionally, we aimed to evaluate the relationship between quantitative measures of modular community structure and the venous ammonia level, Child-Pugh score, and neuropsychological test scores in cirrhosis patients.

## 2. Materials and Methods

### 2.1. Subjects

This study was approved by local institutional review board and was conducted in compliance with Health Insurance Portability and Accountability Act. All subjects gave written informed consent before fMRI or neuropsychologic evaluation. 61 adult patients with cirrhosis (45 males and 16 females; mean age 49.3 ± 10.5 years) were recruited from our inpatient or outpatient departments during June of 2009 and June of 2013. The patients MHE was defined and classified according to the final report of the working party at the 11th World Congresses of Gastroenterology in Vienna in 1998 [16]. The inclusion criteria for recruitment of the patients were as follows: the patients have no clinically proven HE; the patients do not have any MRI contraindication, such as artificial tooth or other foreign bodies in the head causing significant artifacts, which would affect the fMRI exam; all patients had no other diseases affecting brain function, such as drug abuse, psychiatric diseases, and trauma. Thirty-nine age- and gender-matched healthy volunteers were recruited from local community as controls (25 males and 14 females, mean age 49.1 ± 12.8 years). All healthy subjects had neither psychiatric nor neurological history and also no diseases affecting brain structure and functions. Abdominal ultrasound scans revealed no abnormal findings for all healthy subjects. For evaluation of MHE, two typical neuropsychiatric tests, number connection test type A (NCT-A) and digit symbol test (DST), were given to all subjects before MRI studies. A test result was considered abnormal if 2SD is above the mean score of healthy subjects in NCT-A and/or 2SD is below the mean score of healthy subjects in DST. The subject demographics and clinical data are summarized in Table 1.

### 2.2. Laboratory Examinations

Blood biochemistry tests, including prothrombin time, protein metabolism tests (including total protein, globulin, albumin, and the ration of albumin and globulin), bilirubin metabolism tests (including total bilirubin, direct bilirubin, and indirect bilirubin), glutamic pyruvic transaminase, and glutamic oxaloacetic transaminase, were performed within 24 hours before MRI scanning for all patients. Some of the tests above were used to calculate the Child-Pugh score to assess the severity of cirrhosis. The scoring system considered five variables selected by clinical experience, that is, ascites, encephalopathy, prothrombin time, and serum levels of bilirubin and albumin. A score ranging from 1 to 3 was assigned to each variable. Patients were classified into class A (scores 5-6), B (scores 7–9), or C (scores 10–15). Thirteen outpatients did not have the venous blood ammonia test. Laboratory tests were not prescribed to the normal subjects.

### 2.3. Magnetic Resonance Imaging and Preprocessing of fMRI Data

All experiments were performed using a clinical 3T whole-body scanner (TIM Trio, Siemens Medical Solutions, Erlangen, Germany) using a standard birdcage head transmit/receive coil. The head coil was positioned carefully to reduce head movement. A total of 250 volumes of EPI images were obtained axially and the parameters were as follows: field-of-view (FOV) = 240 × 240 mm^2^, matrix size = 64 × 64, flip angle = 90°, TR = 2000 ms, TE = 30 ms, slice thickness = 4 mm, distance factor = 10%, slices = 30. For each subject, a magnetization-prepared, rapid acquisition gradient echo image with isotropic resolution of 1 mm was acquired to obtain high-resolution, T1-weighted anatomical images for spatial normalization. During MRI scans, all subjects were instructed to rest with their eyes closed and heads still.

Resting-state fMRI (rs-fMRI) data were preprocessed by SPM8 (Statistical Parametric Mapping, http://www.fil.ion.ucl.ac.uk/spm/). The first 10 volumes were discarded to allow for T1 equilibration effects. Then, slice timing and realignments were performed on the remaining 240 measures. The time course of head motion was obtained by estimating the translation in each direction and the rotation in angular motion on each axis for all 240 consecutive volumes. Six patients (4 males) and one healthy subject were excluded because either translation or rotation exceeded +1 mm or +1. We also evaluated the differences in translation and rotation of head motion between cirrhotic patients and controls according to the following formula [24]:

(1) Head  motionrotaion =11−L∑i=2L(xi−xi−1)2+(yi−yi−1)2+(zi−zi−1)2,

where *L* is the length of the time series (*L* = 240 in this study) and *x*_*i*_, *y*_*i*_, and *z*_*i*_ are translations/rotations at the *i*th time point in the *x*, *y*, and *z* directions, respectively. The results showed that the two groups had no significant differences (two-sample *t*-test, both *P* > 0.05). The functional data were spatially normalized to the Montreal Neurological Institute (MNI) template and resampled to 3 ∗ 3 ∗ 3 mm^3^. After spatial normalization, the BOLD signal was detrended to abandon linear trend and then filtered (0.01–0.08 Hz) to reduce the effects of low-frequency drift and high-frequency physiological noise. Nuisance covariates including global mean signals, white matter signals, cerebrospinal fluid signals, and head motion parameters were regressed out from the rs-fMRI data. Finally, we obtained mean time series of 90 regions of interests (ROIs) defined by Automated Anatomical Labeling (AAL) atlas [25] for each individual by averaging the rs-fMRI time series over all voxels in each ROI. To demonstrate module changes of functional regions, we mainly computed the functional connectivity networks and their corresponding graph analysis features based on AAL 90 template in this study.

Considering that the range of nodal scales and the difference in template parcellations may result in considerable variation of graph theoretical parameters of functional connectivity networks, we also applied a high-resolution parcellation network with 1024 regions of interest [26] to verify the modularity changes in cirrhotic patients' brain functional connectivity networks.

### 2.4. Functional Connectivity Network and Its Modularity

The Pearson correlation coefficient between any pair of regional time series is computed to form a 90 ∗ 90 matrix of the functional connectivity network. In each subject, the node amount *N* is 90 and the total number of functional connectivities (or edges) is 4005 (*C*_90_^2^ = 90∗89/2 = 4005). Fisher's r-to-z transform is used on the correlation matrix *R*_*ij*_ (*i*, *j* = 1,…, *N*) of each subject to improve the normality of the correlation coefficients [27]. Both positive and negative connections are connectivity in brain network. The absolute value of correlation coefficient is calculated to create the nonnegative matrix *M*_*ij*_ [2]. For simplification, *M*_*ij*_ of each subject is thresholded to create a binarized matrix *A*_*ij*_ in a range of sparsity, comprising between 5% and 10% of the 4,005 possible edges in a network with 90 nodes [28].

In our study, Newman's metric [5] is used as a measure of modularity. The correlation matrices were processed by a code developed by Clauset et al. based on the greedy optimization [29]. Modularity can be regarded as the quantity for measuring the quality of a partition *PA* of a network. If we define the degree *k*_*i*_ = ∑_*j*_ *A*_*ij*_ of a node *i* as the number of connections to the node, the modularity *Q* of each subject can be expressed by

(2) $$\begin{array}{c} Q=\frac{1}{2m}\underset{C\in PA}{\sum }\;\underset{i,j\in C}{\sum }\left(A_{ij}-\frac{k_{i}k_{j}}{2m}\right), \end{array}$$

where *m* is the total edges in the network; the indices *i* and *j* run over the *N* nodes of the graph; the index *C* runs over the modules of the partition *PA*.

To display the functional modules of healthy control, non-HE and MHE groups, the weighted matrix *M*_*ij*_ is first averaged within each group by

(3) MijGroup=1nGroup∑n=1nGroupMijnGroup,

where Group = Control,  non-HE,  or  MHE represents the healthy control, non-HE or MHE group and *n*_Group_ is the total subject number of healthy controls, non-HE or MHE groups. Then, the averaged matrix of each group is thresholded at a sparsity to obtain the binarized matrix _Sparsity_^Group^ *A*_*ij*_. Finally, the module of each group can be achieved by finding the partition having the largest value of _Sparsity_^Group^ *Q* in _Sparsity_^Group^ *A*_*ij*_.

Whenever _Sparsity_^Group^ *Q* of a network reaches maximum, topological principles to each node in group level can be obtained based on the density of intra- and intermodular functional connectivity [30]. Intramodular connectivity is measured by the normalized intramodular degree:

(4) $$\begin{array}{c} z_{i}=\frac{k_{s_{i}}-\bar{k_{s}}}{\sigma_{k_{s}}}, \end{array}$$

where *k*_*s*_*i*__ is the degree connecting the *i*th node to other nodes in the *s*th module, $\bar{k_{s}}$ is the average of *k*_*s*_*i*__ in the module *s*, and *σ*_*k*_*s*__ is the standard deviation of the intramodular degrees in the *s*th module.

Intermodular connectivity can be measured by the participation coefficient:

(5) $$\begin{array}{c} PC_{i}=1-\overset{N_{s}}{\underset{s=1}{\sum }}\frac{k_{s_{i}}}{k_{i}}, \end{array}$$

where *k*_*s*_*i*__ is the intramodular degree as defined above, *k*_*i*_ is the total degree of the *i*th node, and *N*_*s*_ is the number of modules. A node with large *Z* value will have a large number of intramodular connections relative to other nodes in the same module. If a node is linked exclusively to all other modules in the community, its *PC* value will be close to one; if it is extensively linked in its own module to other nodes, its *PC* value will be zero.

Based on the *PC* and *Z* values, nodes of a given functional connectivity network can be partitioned into four categories: connector hub, connector nonhub, provincial hub, and provincial nonhub [2]. A node with *PC* value greater than 0.05 is regarded as a connector node; otherwise, it is a provincial one. A node with *Z* value larger than 1.0 is defined as a hub; otherwise, it is defined as a nonhub. Graphs with different types of nodes are visualized using Pajek (http://vlado.fmf.uni-lj.si/pub/networks/pajek/).

### 2.5. Statistical Analysis

Statistical analysis was performed by using the software SPSS version 13.0 (SPSS Inc. Chicago, IL, USA). One way analysis of covariance (ANCOVA) with age and gender as covariates was used to analyze the difference of the modularity measure *Q* among healthy controls, non-HE and MHE patients. Post hoc comparisons (Sidak-corrected) were performed between every two groups at each sparsity. Correlations between *Q* values and the venous blood ammonia level, Child-Pugh score, and neuropsychological test scores were calculated. All data were shown as mean ± SD. A *P* value less than 0.05 was considered statistically significant.

## 3. Results

### 3.1. Demographical and Clinical Data

Fifty-five patients (43 males and 12 females, mean age, 49.1 ± 10.1 years) and 38 controls (26 males and 12 females, mean age, 48.6 ± 12.7 years), matched for age (*P* = 0.995, two-sample *t*-test) and gender (*P* = 0.216, Mann-Whitney *U* test), were included for further analysis. Healthy subjects had a NCT-A score of 45.1 ± 12.7 s and a DST score of 49.8 ± 9.8. These scores were used to differentiate MHE patients from non-HE patients. Thirty-one patients (24 males and 7 females, mean age, 46.6 ± 10.2 years) with normal NCT-A scores and DST scores were classified as non-HE patients and twenty-four patients (19 males and 5 females, mean age, 52.3 ± 9.2 years) with abnormal NCT-A or DST test scores were identified as MHE (Table 1). No correlation was found between the venous blood ammonia level, Child-Pugh score, and the neuropsychiatric tests (both *P* > 0.05) in cirrhotic patients.

### 3.2. The Modularity *Q* Values of Healthy Control, non-HE and MHE Patients

**Figure 1:**
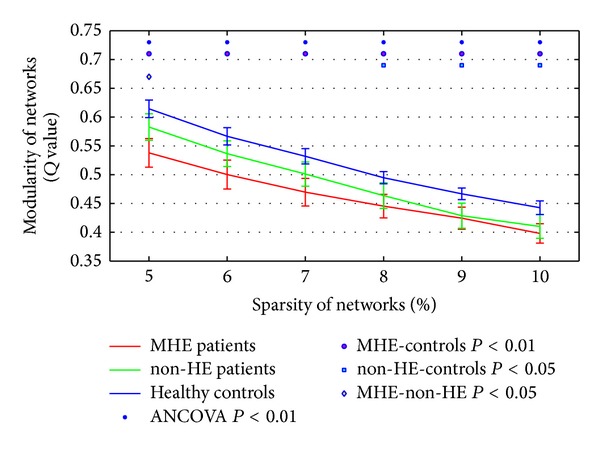
The network modularity *Q* values of healthy controls, non-HE and MHE patients from sparsity of 5% to sparsity of 10% at 1% intervals.

The modularity *Q* values of healthy control, non-HE and MHE patients were illustrated from sparsity of 5% to sparsity of 10% at 1% intervals in Figure 1. One-way ANCOVA results showed significant differences in the *Q* value among healthy controls, non-HE and MHE patients (*P* < 0.01). For three groups, the *Q* value declined monotonically as a function of increasing sparsity; that is, maximum modularity would be greatest for networks with highest sparsity. Post hoc analysis showed that the modularity of brain networks in MHE patients was significantly lower than those in healthy controls over the entire range of sparsity (*P* < 0.01). Moreover, the modularity of non-HE patients was markedly lower than controls at 8% ≤ sparsity ≤ 10% (*P* < 0.05). In addition, the modularity of MHE patients was significantly lower than non-HE patients only at sparsity of 5% (*P* < 0.05). Correlation coefficients between the modularity *Q* values and the venous blood ammonia level, Child-Pugh score, and neuropsychological test scores were shown in Table 2. The *Q* values were positively correlated with NCT-A scores in all selected sparsity and were negatively correlated with DST score at 5% ≤ sparsity ≤ 7%. No correlation was found between *Q* values and the venous blood ammonia level and Child-Pugh score (all *P* > 0.05).

We also verified the modularity *Q* values of three groups using 1024 template (see Supplementary Figure 1 available online at http://dx.doi.org/10.1155/2014/727452). The modularity results based on high-resolution parcellation were consistent with our findings using AAL 90 template.

### 3.3. The Total Number of Modules and the Selection of the Sparsity of Functional Connectivity Networks

**Figure 2:**
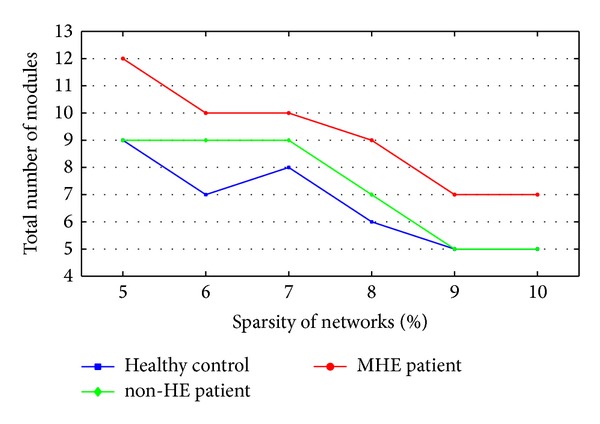
The total numbers of modules of healthy controls, non-HE and MHE groups from sparsity of 5% to sparsity of 10% at 1% intervals.

Figure 2 shows the total number of modules of each group's mean network over the entire range of sparsity. The modules of three groups' mean networks nearly decreased as a function of increase in connection density. The number of modules in the MHE group was greater than that in the non-HE and healthy control group over the entire sparsities. The number of modules of non-HE group was higher than that of controls at 6% ≤ sparsity ≤ 8%.

Salvador et al. suggested that 90 cortical and subcortical regions could be partitioned by cluster analysis into 6 major systems of anatomically and functionally related regions in healthy subjects [30]. If the density is high, many small modules might immigrate into a large module. At 9% ≤ sparsity ≤ 10%, there were only five modules in our healthy controls which would limit the illustration of modules of three groups. Hence, low connection densities, for example, sparsity ≤ 8%, should be chosen to calculate the modules. However, if the density is too low, the binary networks would be too sparse to ensure the full connection of nodes. In normal subjects, the brain network was a fully connected one [2, 29, 30]. We found that there were isolated nodes in our healthy controls when sparsity ≤ 7%. Considering the reasonable number of modules and connection of nodes in our healthy subjects, we mainly displayed the modules of healthy control, non-HE and MHE groups at sparsity = 8%, where our healthy control group had the same number of modules as that reported by Salvador et al. [30]. To show the consistent results at different thresholds, we also illustrated the modules of three groups at sparsities of 7% and 9%.

### 3.4. Modules Changes in Healthy Control, non-HE and MHE Groups

**Figure 3:**
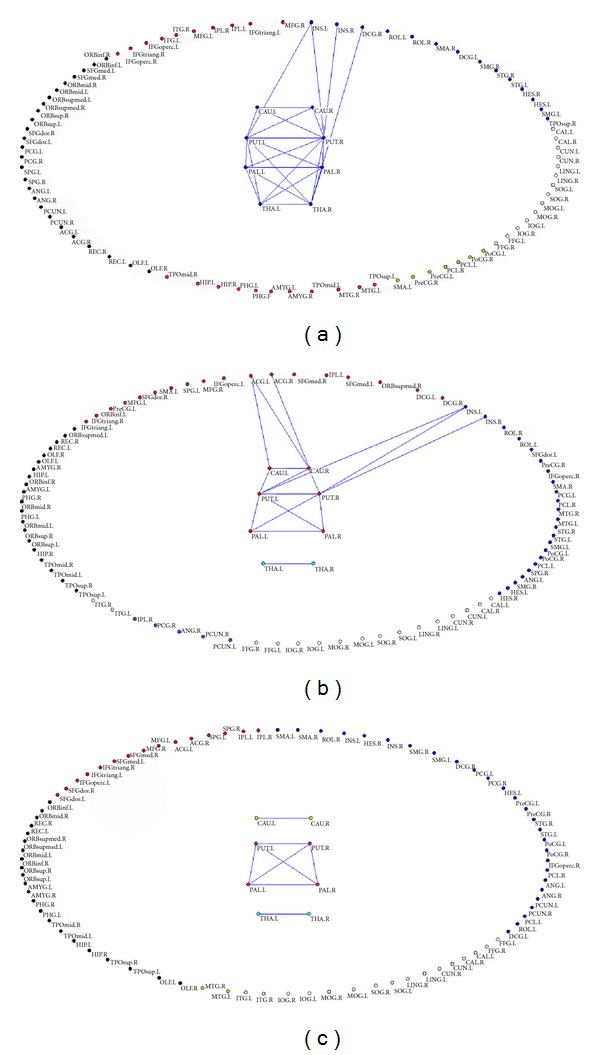
The community structures of healthy control, non-HE and MHE groups for mean functional networks at sparsity of 8%. (a) The community structure of healthy control group; (b) the community structure of non-HE group; (c) the community structure of MHE group.

Figure 3 shows the community structures for three groups' brain functional networks at sparsity = 8%, respectively. To focus on the connectivity changes between cortical and subcortical regions and on the module changes of subcortical regions, only connections between cortical and subcortical regions and between subcortical regions were shown.

The healthy brain functional network comprised 6 connected modules at sparsity = 8%, which varied in size from 26 to 7 regional nodes (Figure 3(a)). Eight subcortical regions (e.g., bilateral caudate, putamens, pallidum, and thalami) were included in the second largest module containing 22 regions. Twenty-four edges were found between cortical and subcortical regions and between subcortical regions. Among 24 connections, 4 connectivities were found between cortical and subcortical regions, such as the connections between left insula and bilateral putamens, right insula and right putamen, and between right middle cingulum gyrus (DCG) and right thalamus; 20 ones were between subcortical regions, for example, the connections between left and right caudate, bilateral caudate and bilateral putamens, left and right putamen, bilateral putamens and bilateral pallidum, left and right pallidum, bilateral putamens and bilateral thalami, bilateral pallidum and bilateral thalami, and left and right thalamus.

The non-HE group had 7 modules at sparsity = 8%, among which bilateral thalami were isolated as an unconnected module (Figure 3(b)). There was a connection between left and right thalamus, while there was no connection between thalamus and other brain regions. Basal ganglia were involved in the largest module with 24 region nodes. Sixteen connections were detected between cortical regions and basal ganglia and between subregions of basal ganglia. Among 16 connections, 6 edges were between cortical regions and basal ganglia, such as the connections between left anterior cingulate gyrus (ACG) and bilateral caudate, right ACG and right caudate, left insula and bilateral putamens, and right insula and right putamen; 10 ones were between subregions of basal ganglia, such as the edges between left and right caudate, left and right putamen, left and right pallidum, bilateral putamens and bilateral pallidum, left caudate and left putamen, and right caudate and bilateral putamens.

In the MHE group, 9 modules were found at sparsity = 8% (Figure 3(c)). The subcortical region nodes were partitioned into three isolated modules and they were the caudate module composed of bilateral caudate, the thalamus module composed of bilateral thalami, and the putamen/pallidum module comprising bilateral putamens and pallidum. Among subcortical nodes, there were connections between left and right caudate, left and right thalamus, and bilateral putamens and pallidum. No connectivity was found between cortical and subcortical nodes.

### 3.5. Changes of Node Roles in Healthy Control, non-HE and MHE Groups

**Figure 4:**
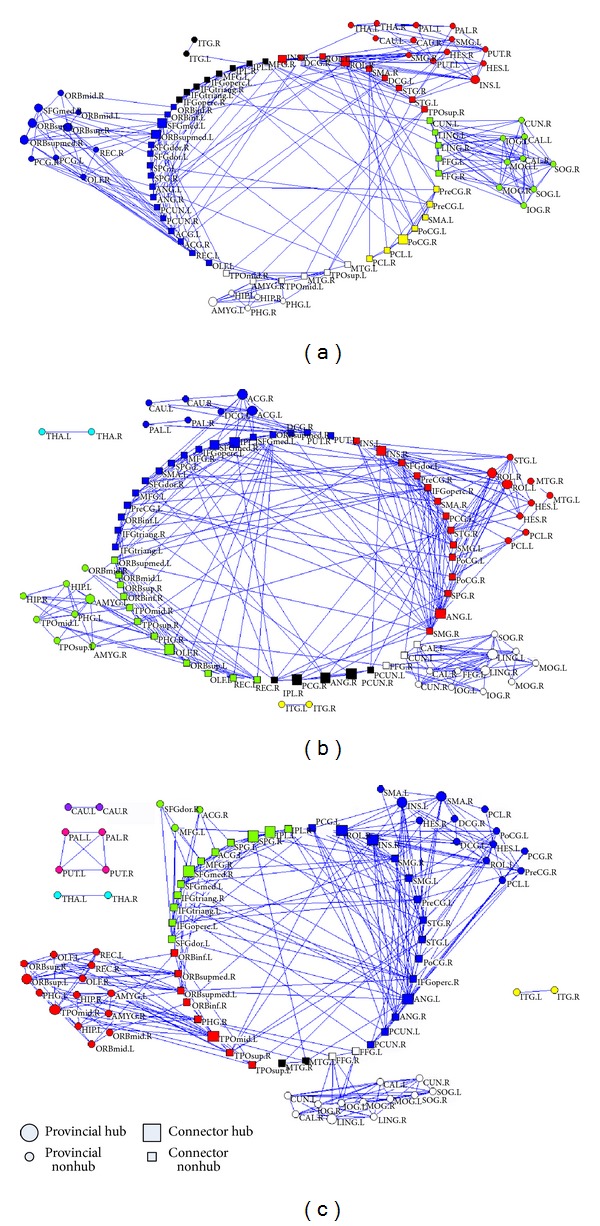
The node roles of healthy control, non-HE and MHE groups. (a) The node role of healthy control group; (b) the node role of non-HE group; (c) the node role of MHE group.

The node roles of healthy control, non-HE and MHE groups were displayed by connector hub, connector nonhub, provincial hub, and provincial nonhub and all connections between nodes were shown at sparsity = 8% (Figure 4).

In both healthy control and non-HE groups' mean networks (Figures 4(a) and 4(b)), over half of the whole-brain regions (51/90) were connector nodes which had numerous connections to other modules, while only 38 regions were connector nodes in MHE group (Figure 4(c)). The connector coefficients for healthy control, non-HE and MHE groups were 56.7% (51/90), 56.7% (51/90), and 42.2% (38/90), respectively. The total number of hubs in non-HE group (Hubs = 15) was greater than those in healthy control group (Hubs = 11) and in MHE group (Hubs = 12). Eight of the 15 hubs in non-HE group were categorized as connector hubs and 7 as provincial hubs, but only 5 connector hubs/7 provincial hubs in the healthy controls and 7 connector hubs/5 provincial hubs in the MHE group.

To verify our findings about the altered modular structures in cirrhotic patients, we also demonstrated the modules of three groups at the 7% and 9% sparsities (Supplementary Figures 2 and 3). The modules at the 7% sparsity were very similar to those at 8% (Supplementary Figure 2). However, there was one isolated module in the healthy group. When the network sparsity was 9%, isolated modules in subcortical regions were also found in MHE patients (Supplementary Figure 3). However, modules with different brain functions were merged into large one, indicating that the brain regions were not well partitioned.

## 4. Discussion

The present study showed (1) significantly decreased modularity of functional brain networks in cirrhotic patients, which depended on the severity of HE and was associated with neuropsychological test scores; (2) altered functional connectivity between cortical and subcortical modules, less intrasubcortical connection, and more isolated modules, related to the development of MHE; and (3) unchanged numbers of connect nodes and increased total hubs in non-HE patients.

Disrupted modularity of large-scale functional brain networks in cirrhotic patients within modules, reflected by decreased *Q* value, is expected because dysmetabolic neurotoxins were accumulated in patients' brains, resulting in the swelling of astrocytes and abnormal communication between neurons [31]. Since the modularity *Q* value is defined by the difference between the fraction of edges within each module and those random edges without regarding the community structure, it is able to represent the property of the connectivity structure within modules [5]. Networks with low modularity trend to be random graphs [2]. And hence, low *Q* values in cirrhotic patients, especially in MHE patients, supported that their functional connectivity networks lost self-organization properties within functional modules. Since the high clustering of connections between nodes in the same module will favor low wiring cost, modular networks may be of the property of small-worldness which is advantageous for nervous system design [32]. Previous small-world study in cirrhotic patients showed that small-worldness might decrease with the progression of HE [33]. Our modularity results supported that functional connectivity networks in cirrhotic patients were impaired in large-scale. Also, we found quantitative *Q* value correlated with neuropsychological test scores rather than with Child-Pugh score and venous blood ammonia level. Frontal dysfunction in MHE patients, such as attention [19, 21] and working memory impairment, has been consistently reported [34]. Our finding is consistent with these studies and indicates cognitive dysfunction can be associated with disrupted functional connectivity within modular structures in cirrhotic patients. Taken together, these support our disconnect hypothesis of HE; that is, disrupted regional brain connectivity network can be involved in the cognitive dysfunction in cirrhotic patients.

More modules in cirrhotic patients were found compared with those in controls, indicating that cirrhotic patients lost functional connectivity between modules. In particular, subcortical modularity loss, disrupted connections between cortical and subcortical modules, and less intrasubcortical connections occurred during the progression of the disease. Particularly in MHE patients, functional connectivity between cortical and subcortical modules was disrupted, resulting in more isolated modules in these patients. These findings of more localized community structure in cirrhotic patients than in controls have been reported in previous literatures on aging, schizophrenia, epilepsy, and chronic back pain [2, 10–15]. We further found that more modules in cirrhotic patients can be associated with basal ganglia-thalamocortical circuit disruption because of lost community structure between these more localized subcortical communication structures (caudate nucleus, basal ganglia, and thalami) in MHE patients compared with healthy controls and non-HE patients, which depended on the severity of the disease as shown in Figure 3. This finding supported the important role of basal ganglia-thalamocortical circuit in the development of MHE [18]. Disrupted module organization of basal ganglia-thalamocortical circuit can be one of the key causes of more modules in cirrhotic patients than in controls. Histopathologically, Alzheimer type II astrocytes, the characteristic neuropathologic findings in cirrhotic patients, are predominantly found in the cortex, putamen, pallidum, and caudate nucleus [35]. Radiologically, the symmetrical hyperintensity of basal ganglia on T1-weighted images is often observed [36]. Recently fMRI studies indicated abnormal resting-state functional connectivity of this circuit in MHE patients [22, 23]. Disrupted effective connectivity network of the basal ganglia, anterior cingulate cortex, and striatum was also reported [37]. The decreased functional connectivity between thalamus, many cortices, and basal ganglia indicated reduced integrity of thalamic resting-state network in MHE. Taken together, we believe that disrupted modular structure and functional connectivity of basal ganglia-thalamocortical circuit could result in development of MHE.

Our study indicated non-HE patients had abnormal communication structures. The abnormal modularity findings in the non-HE patients included slightly decreased *Q* value and slightly increased modules. Importantly, in non-HE patients, the numbers of connect nodes did not change while the total numbers of hubs increased, indicating that there could be a compensation for the decreased modularity in their functional connectivity networks. Although more modules were found in non-HE patients compared with healthy subjects at the same sparsity, unchanged numbers of connect nodes could ensure the normal communications between functional modules. More connector and provincial hubs were found in non-HE patients, suggesting that more hubs were needed to make up the degradations of functional connectivity both between and within modules. In MHE patients, the numbers of connect nodes greatly decreased, which might not be sufficient for the basic information transmission between modules, explaining why more isolated modules were found in MHE patients. MHE patients were of less connector and provincial hubs compared with non-HE patients, indicating that their brain networks were seriously impaired and could not make up the normal communications between modules by increasing hubs.

We did not find correlation between the venous blood ammonia level and Child-Pugh score with modularity *Q* values in three groups, which could be related to the cirrhotic patients recruited in our study. Since only cirrhotic patients without overt HE were included in this study, the ammonia level and Child-Pugh score in our patients were not very high, which might make it difficult to show the relationships between the factors and the modularity *Q* values. Future studies should include the cirrhotic patients with overt hepatic encephalopathy.

### 4.1. Limitations

There are still some limitations in our study. First, the sample size of patients with minimal HE was small and this would affect the validity of the statistical analysis of this preliminary study. Thus, a large-cohort study is needed. However, since a standard statistical processing pipeline was followed with accepted software and procedures in this study, we believe most findings are rational based on these analyses. Second, this study was not longitudinal and overt HE patients were not included in this study. Therefore, we cannot draw a conclusion on the progression pattern of brain functional connectivity modularity from MHE to overt HE, which needs to be further investigated. Additionally, no significant correlations were found between liver functions and the modularity *Q* values in this study, which might also be related to the selection of our patients because no overt HE patients were included in this study. Third, we used two neuropsychiatric tests to evaluate MHE which were recommended by the working party at the 11th World Congresses of Gastroenterology. Whole battery of neuropsychiatric tests should be performed in future study. Fourth, although we tried to pick up a reasonable threshold to demonstrate our findings, it is rather arbitrary for the selection of thresholds. Hence, we suggested that different thresholds should be tested to find if results are sensitive to them.

## 5. Conclusions

In conclusion, our study demonstrated that cirrhotic patients had disrupted modularity of functional brain networks associated with neurocognitive dysfunction, in accordance with the severity of HE. Subcortical modularity loss, disrupted connection between cortical and subcortical modules, and less intrasubcortical connection, especially, in basal ganglia-thalamocortical circuit were found with the development of MHE. Adjustment of hub and provincial nodes could be a compensation for the disrupted modularity in non-HE patients.

## Supplementary Material

Different ranges of nodal scales and template parcellations may result in considerable variation of graph theoretical parameters of functional connectivity networks. Hence, we varfied our results on a high-resolution parcellation network with 1024 regions of interest [26]. Changes in Q values based on high-resolution parcellation network (See Supplementary Figure 1) were consistent with those on AAL 90 template (See Figure 1). In our study, we mainly displayed our results at 8% network sparsity (See Figure 3 and 4). We also showed the modules of three groups at the 7% and 9% sparsities (See Supplementary Figure 2 and 3). Our module results at three differe sparsities were quite similar, indicating that they were not sensitive to the selection of thresholds.

## Figures and Tables

**Table 1:** Demographical and clinical data of the healthy controls and cirrhotic patients.

Variable	Healthy controls (*n* = 38)	non-HE patients (*n* = 31)	MHE patients (*n* = 24)	*P* value
Age (Y)	48.6 ± 12.7	46.6 ± 10.2	52.3 ± 9.2	0.187^∣^
Gender (M/F)	25/13	24/7	19/5	0.411^~^
NCT-A (s)^∗#^	45.1 ± 12.7	42.7 ± 9.6	69.5 ± 17.4	**<0.00**1^∣^
DST (score)^∗#$^	49.8 ± 9.8	42.5 ± 9.6	25.6 ± 7.7	**<0.00**1^∣^
Posthepatitic cirrhosis (*n*)	—	21	17	—
Biliary cirrhosis (*n*)	—	3	3	—
Schistosomal cirrhosis (*n*)	—	0	1	—
Alcoholic cirrhosis (*n*)	—	1	1	—
Budd-Chiari syndrome (*n*)	—	1	0	—
Unknown aetiology (*n*)	—	5	2	—
Child-Pugh scores (score)	—	6.4 ± 1.5	6.8 ± 1.7	0.40^!^
Child-Pugh scale (A/B/C)	—	20/10/1	11/12/1	—
Ammonia level (umol/L)^@^	—	52.8 ± 34.6 (*n* = 26)	61.2 ± 28.8 (*n* = 19)	0.398^!^

Values are mean ± SD or number of patients; MHE: minimal hepatic encephalopathy; NCT-A: number connection test type A; DST: digit symbol test.

^@^Ammonia is obtained in 19 MHE patients and 26 non-HE patients.

^!^stands for the results of two-sample *t*-test.

^∣^stands for the result of the one-way ANOVA.

^~^stands for the result of the Chi-Square test.

*stands for significant difference between MHE and non-HE patients (post hoc *P* <0.05, Bonferroni-corrected).

^#^stands for significant difference between MHE patients and controls (post hoc *P* <0.05, Bonferroni-corrected).

^$^stands for significant differences between non-HE patients and controls (post hoc *P* <0.05, Bonferroni-corrected).

**Table 2:** Correlations between the modularity *Q* values and the venous ammonia level, Child-Pugh score, and neuropsychological test scores.

Sparsity	5%	6%	7%	8%	9%	10%
NCT-A (s)^a^	0.393***	0.379***	0.341***	0.330**	0.283**	0.339***
DST (score)^a^	−0.342***	−0.288**	−0.250*	−0.194	−0.148	−0.201
Child-Pugh score^b^	−0.072	−0.086	−0.078	−0.022	0.034	0.035
Ammonia level (umol/L)^b^	0.050	−0.006	−0.051	0.054	0.065	0.047

NCT-A: number connection test type A; DST: digit symbol test.

^a^Correlations were performed in all subjects.

^b^Correlations were performed in cirrhotic patients.

*P < 0.05, **P < 0.01, and ***P < 0.001.

## Abbreviations

NCT-A: Number connection test type A
DST: Digit symbol test
MHE: Minimal hepatic encephalopathy
OHE: Overt hepatic encephalopathy
non-HE: Cirrhotic patients without minimal hepatic encephalopathy.
