# Supplementary material for: Altered Modular Organization of Functional Connectivity Networks in Cirrhotic Patients without Overt Hepatic Encephalopathy
Source: Biomed Res Int. 2014 Jun 5;2014:727452. doi: 10.1155/2014/727452 (PMC4066720; doi:10.1155/2014/727452)
Supplement: Supplementary file 1 — Different ranges of nodal scales and template parcellations may result in considerable variation of graph theoretical parameters of functional connectivity networks. Hence, we varfied our results on a high-resolution parcellation network with 1024 regions of interest [26]. Changes in Q values based on high-resolution parcellation network (See Supplementary Figure 1) were consistent with those on AAL 90 template (See Figure 1). In our study, we mainly displayed our results at 8% network sparsity (See Figure 3 and 4). We also showed the modules of three groups at the 7% and 9% sparsities (See Supplementary Figure 2 and 3). Our module results at three differe sparsities were quite similar, indicating that they were not sensitive to the selection of thresholds. [file 727452.f1.pdf]

Supplementary materials

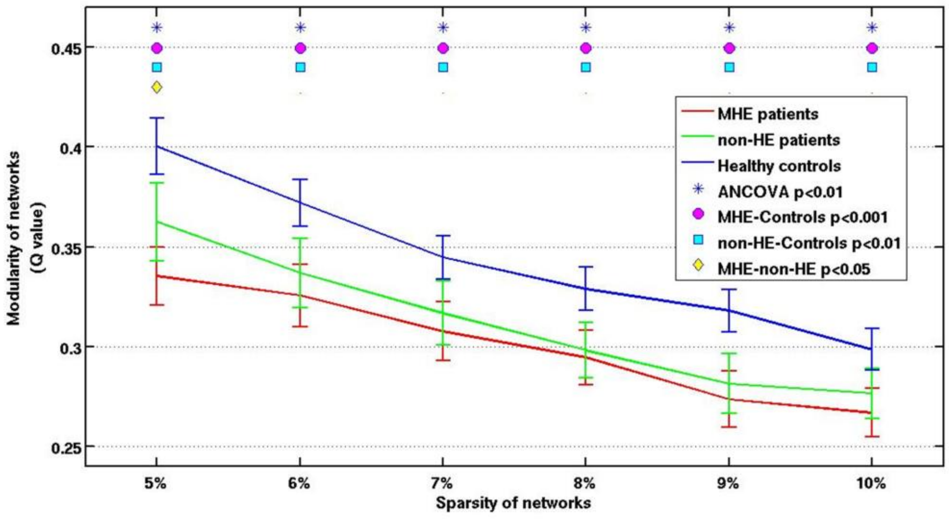

Supplementary figure 1 The network modularity Q values of healthy controls, non-HE and MHE patients from sparsity=5% to sparsity=10% at 1% intervals.(Based on1024 parcels)

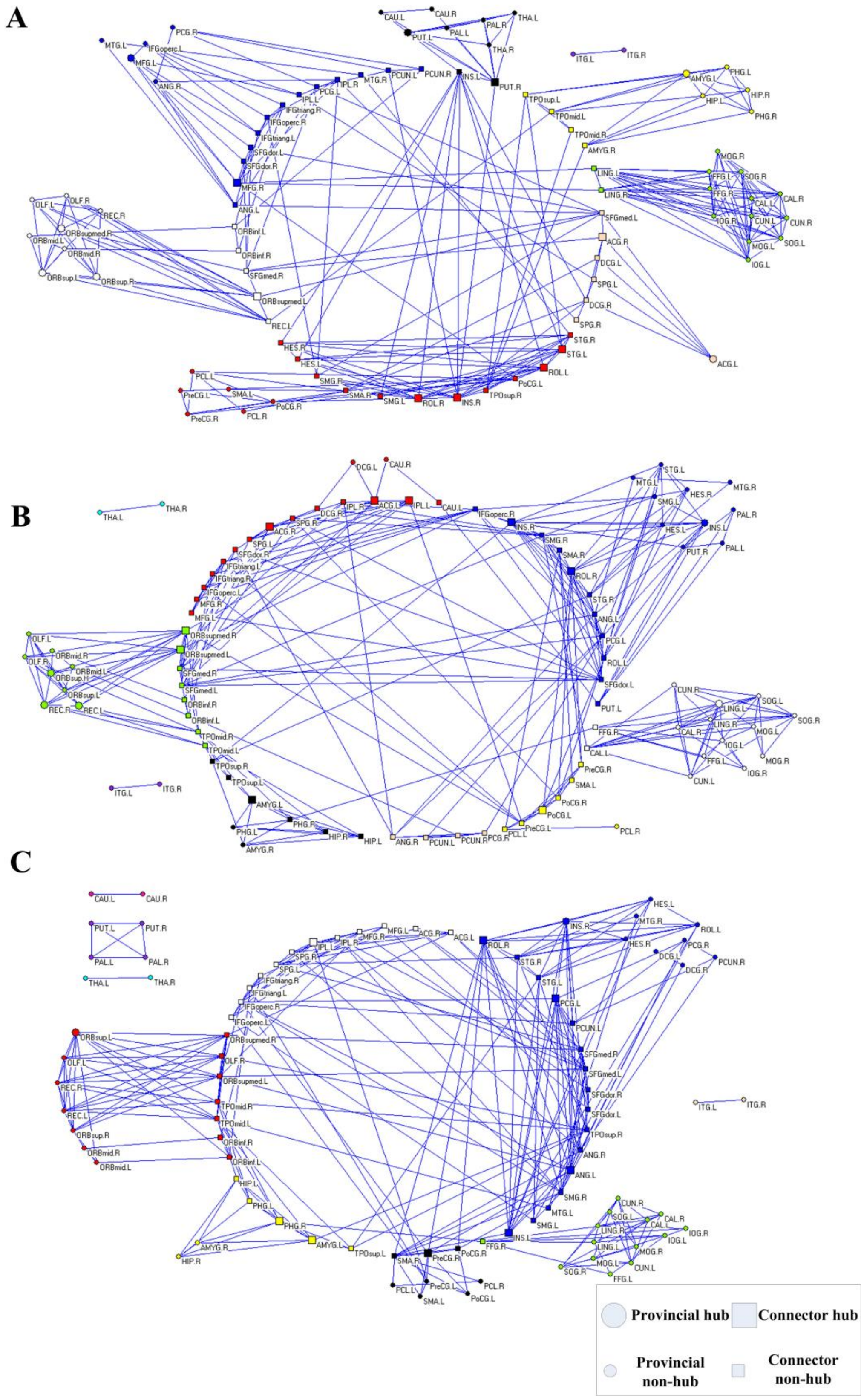

Supplementary figure 2 The community structures of healthy control, non-HE and MHE groups' for mean functional networks at sparsity=7%. A. The community

structure of Healthy control group; B. The community structure of non-HE group; C. The community structure of MHE group.

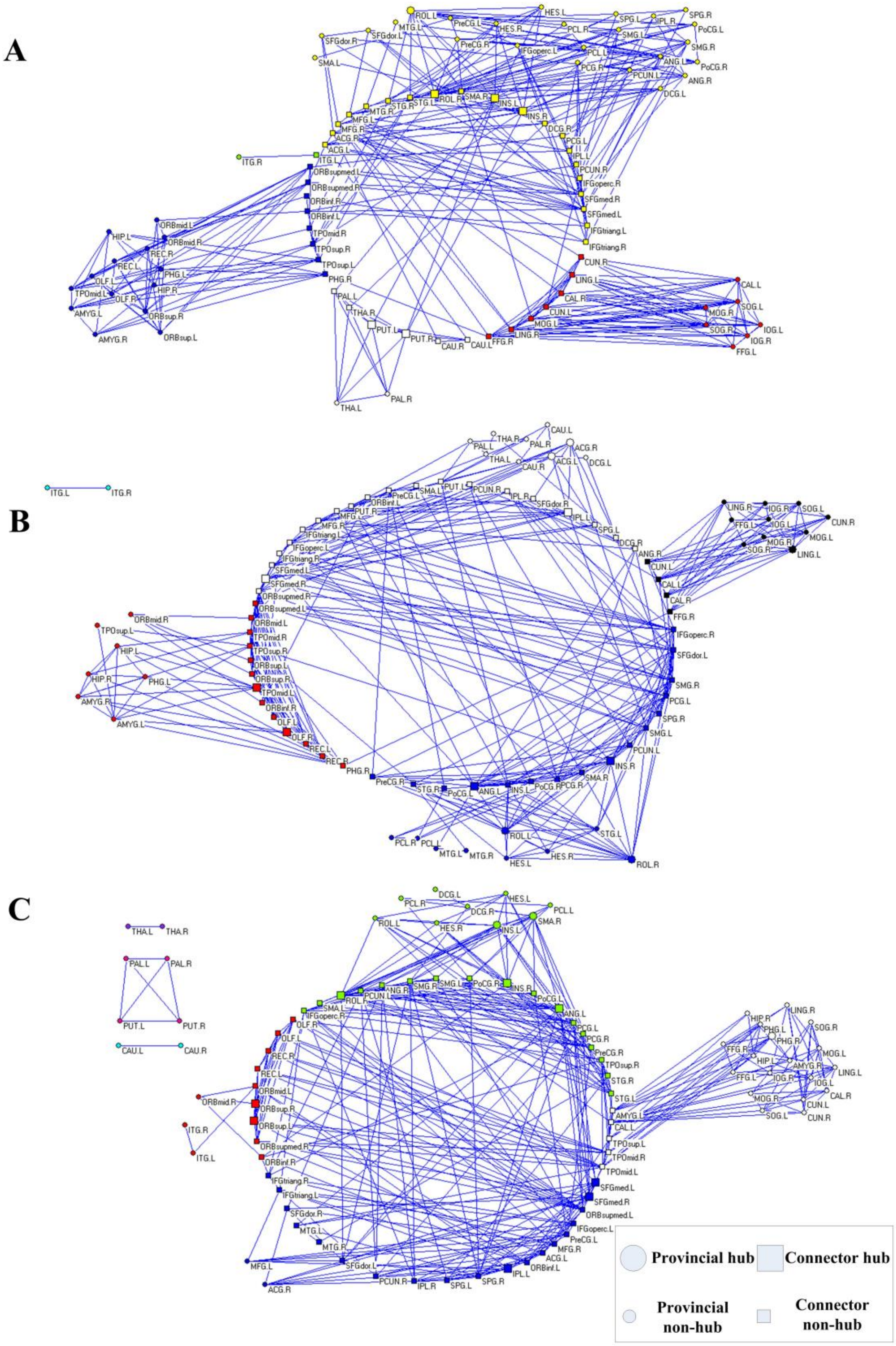

Supplementary figure 3 The community structures of healthy control, non-HE and MHE groups' for mean functional networks at sparsity=9%. A. The community

structure of Healthy control group; B. The community structure of non-HE group; C. The community structure of MHE group.
